# Book Review

**Published:** 1896-03

**Authors:** 


**Medical Partnerships, Transfers, and Assistantships.** By William Barnard and G. Bertram Stocker. Pp. xi., 249. London: Stevens and Sons, Limited. 1895.

In the preface of this eminently useful work the authors call attention to the fact that no text-book dealing with the subject here treated has ever been published before, and that each draftsman has had to evolve a scheme out of his own brain, unaided by experience or knowledge of the customs of our profession. No one now can blame anyone but himself if the solicitor, who draws up his agreement, fails to fully protect him from the wiles of a dishonest partner or principal, or from the accidents and differences which may at any time occur in the course of a partnership. To a medical mind the book is stiff reading; for legal phraseology is not the easiest in the world, nor are the decisions of judges always expressed in the clearest language.

In Part I., Mr. Stocker's wide experience as Managing Director of the Scholastic, Clerical, and Medical Association finds scope in giving the soundest advice to intending partners or assistants—unless that which *Punch* gave to those about to marry is wisest of all. The various forms for agreements, etc., are excellent, and appear to cover any exigencies which may arise. In Part II., the Partnership Act of 1890 is given in full, with very useful notes and precedents, which we believe are so dear to the legal mind; being followed by a long chapter on goodwill and restrictions to practice, an appendix of cases on restrictive covenants, and other chapters on introductions and assistants. Both the index and the table of cases referred to are very complete.

We can heartily recommend this book to intending partners, and to their solicitors; and we are sure that if the sound advice given in it is followed, we should hear less of the disappointment and disagreements that so often arise in medical partnerships. We hope every solicitor in the country will supply himself with a copy.

**The Animal Tuberculoses, and their Relation to Human Tuberculosis.** By Ed. Nocard. Translated by H. Scurfield, M.D. Pp. 143. London: Baillière, Tindall and Cox. 1895.—The great interest of this book lies in the demonstration of the importance of contagion and the insignificance of heredity in the propagation of bovine tubercle; and the translator observes that if the children of tuberculous parents were protected from infection by cohabitation or ingestion, the importance of heredity as a cause of the disease, or even of the predisposition to it, would dwindle away into insignificance.

**Myxœdema and the Thyroid Gland.** By John D. Gimlette. Pp. vii., 128. London: J. & A. Churchill. 1895.—This is a thesis for the Medical Degree of the Lisbon Medical School amplified. It is essentially a compend of the various writings on Myxœdema, and, as the writer has been industrious in consulting his authorities and has made his book as complete a *resumé* as possible of the existing state of knowledge on the subject, it forms a useful compilation. He has brought it thoroughly up to date and gives the latest views as to treatment. There is a good index, a necessity in a work of this kind.

**On the Relation of Diseases of the Spinal Cord to the Distribution and Lesions of the Spinal Blood Vessels.** By R. T. Williamson, M.D. Pp. 43. London: H. K. Lewis. 1895.— In this we have a reprint, with some additions, of papers which were published in the *Medical Chronicle.* It is well written, is illustrated with numerous drawings, and forms a valuable contribution to a subject which has recently attracted much attention. The author brings forward many new facts and adduces the observations of other writers to show that the pathological changes in diseases of the spinal cord often bear a remarkable anatomical relation to the distribution of the spinal vessels; and further, that well-marked morbid changes in the vessels themselves are of frequent occurrence. We think that he successfully demonstrates that vascular lesions play a larger part in the production of spinal cord disease than has hitherto been supposed. The book, besides containing much original observation, gives full references to the chief work on this subject by other observers and will repay careful perusal.

**The Conditions of Radical Cure in Cancer** [and other Papers]. By HERBERT SNOW, M.D. Pp. 63. London: J. & A. Churchill. 1895.—This small volume consists of reprints of four lectures on cancer by Dr. Snow. One third of the pages is taken up by short and incomplete reports of cases; of the remainder the major part is devoted to such banal themes as advocating early operation and excision of glands whether obviously diseased or not. There is an inadequate discussion of what the author calls "fibroma of adolescence," which he says is "largely due to direct pressure;" he also makes the statement that "something must also be ascribed to indirect pressure effects and to sympathy with the uterus, also struggling for unimpeded development"! How these remarkable causes bring about a "fibroma" of the breast, which disappears under inunction of a lead iodide ointment, is not explained. We cannot recommend any member of our profession to buy the book; but it is eminently fit for the railway book-stall.

**Early Scoliosis or Curable Curvatures of the Spine.** By PERCY G. LEWIS, M.D. Pp. 49. London: John Bale & Sons. 1895. — By this publication the author wishes to assist practitioners in the diagnosis and treatment of cases of lateral curvature when seen at an early stage, and we think it will well serve such a useful purpose. It is mainly clinical, but an outline of the pathological changes met with in these cases is also given, while practical directions as to treatment are added, rendering the work as complete as it can be in such a small compass. The author does not approve of mechanical supports and especially objects to the use of Sayre's jackets, but he recommends various gymnastic exercises (which are fully described and figured) and a special form of "spinal cushion," which he describes as a kind of vertical bolster fixed to the back of the chair on the side of the raised shoulder. He also recommends a "spinal chair," the back of which moves on a pivot, in such a way that either margin may be made to advance while the other recedes. We have no doubt that when properly used these appliances are valuable, but we entirely differ from the author when he signifies his disbelief in the use of massage combined with Swedish exercises. We would far rather trust to a combination of these than to "the author's spinal cushion" or "the author's spinal chair."

**Some Points in the Practice of Lithotrity or Litholapaxy.** By REGINALD HARRISON. Pp. 16. London: John Bale & Sons. 1895.—We have here a lecture delivered at a post-graduate course, which contains some excellent hints as to the successful management of this operation. It may be read with advantage by all who wish to do the operation.

**A Handbook of the Diseases of the Eye and their Treatment.** By HENRY R. SWANZY, M.B. Fifth Edition. Pp. xviii., 582. London: H. K. Lewis. 1895.—This well-known work maintains its reputation amongst the very best guides to a knowledge of diseases of the eye. Dr. Louis Werner has been associated with the author in editing this last edition, which has been enlarged and to some extent re-arranged. The book has been kept well up to date, and in making reference to it for any new or abstruse point in ophthalmic practice which is of real practical value one is seldom disappointed. Among other chapters of great excellence may be mentioned that on "Amblyopia and Amaurosis due to Central and other Causes," in which the various defects of vision that depend upon impairment of the various tracts and centres of the nervous system are fully and ably discussed. This is a subject of interest to the physician as well as to the ophthalmic surgeon. All parts of the book are well and concisely written.

**Medical Electricity.** By H. LEWIS JONES, M.D. Being the Second Edition of *Medical Electricity*, by W. E. Steavenson, M.D., and H. Lewis Jones, M.D. Pp. xiii., 474. London: H. K. Lewis. 1895.—We are glad to see that this excellent manual has reached a second edition. It is to be thoroughly recommended, as it gives just the information most useful to the medical practitioner to enable him to apply electricity to his cases, to select the cases in which this treatment will be beneficial, and that form of it which will give the best results. The book is a thoroughly practical one, and from it a good working knowledge of medical electricity may be gained. This edition retains the main features of the first, but the earlier chapters have been simplified in order to make them more generally useful.

**Manual for the Church Lads' Brigade Medical Staff Corps.** Pp. 71. London: The Church Lads' Brigade. 1895.—This is an accurate and fairly complete little manual, giving instruction in first aid to the wounded. The directions are clear and sufficiently full, since they are intended to be supplemented by oral instruction, but more definite warning should, we think, be given against allowing patients with fractures of the lower limb to attempt to stand, on the care of varicose veins, and against giving stimulants to epileptic patients. A large portion of the little book is taken up with directions for stretcher drill and the precautions to be observed in carrying the wounded. The manual has been compiled with much care so far as it goes.

**Hygiene and Public Health.** By LOUIS C. PARKES, M.D. Fourth Edition. Pp. 531. London: H. K. Lewis. 1895.—This excellent students' manual retains its place as a concise and useful text-book of Public Health. In the fourth edition some slight additions are made and the information is brought up to date.

**Bread, Bakehouses, and Bacteria.** By F. J. Waldo, M.D. and David Walsh, M.B. Pp. 65. London: Baillière, Tindall and Cox. 1895.—This collection of papers deals with the necessity for the proper regulation of bakehouses, having regard not only to the interests of the consumer but also of the workman. While the authors appear to attach somewhat undue importance to their bacteriological investigations, and, as in Chap. IV. (Cholera in Bread), seem to strain somewhat for effect, the general practical conclusions of the papers are unassailable.

**The Truth about Vaccination.** By Ernest Hart, D.C.L. Second Edition. Pp. iv., 59. London: Smith, Elder & Co. 1895.—The case for vaccination is here fairly and concisely stated. There is very great advantage in having for reference a book wherein, without excessive labour, the answers to the chief anti-vaccination arguments may be found; and every practitioner would do well to obtain and study a copy of this work.

**A Summary of the Vital Statistics of the New England States for the Year 1892.** Pp. 59. Boston: Damrell & Upham. London: P. S. King & Son. [1895.]—The neglect of registration in the State of Maine rendered impossible any previous attempt at classification of the statistics of the New England States as a whole. This summary is carefully and clearly compiled, and forms an excellent beginning of systematic vital statistics for the New England States.

**The Johns Hopkins Hospital Reports,** Vol. IV., No. 7—8. *Report in Gynecology*, III. Baltimore: The Johns Hopkins Press. 1895.—This report contains articles on hydrosalpinx, post-operative septic peritonitis and tuberculosis of the endometrium. More than half the book is taken up by the first of these subjects, which is classified under the heads of (1) hydrosalpinx simplex; (2) hydrops tubæ profluens; (3) hydrosalpinx follicularis; (4) tubo-ovarian cyst. It will be seen that this is an ordinary classification with the addition of hydrosalpinx follicularis, which is a new variety. Briefly it may be said that this last does not differ externally from hydrosalpinx simplex, but on section it presents a honeycombed appearance, and may or may not show a central lumen. In fact it resembles a cystic form of endo-salpingitis follicularis described by Martin and Orth. Short histories of twenty-seven cases of hydrosalpinx are given. The second part of the work was inspired by four deaths from abdominal section occurring about the same time, and apparently due to the use of an infected gut. Tuberculosis of the endometrium is thought to be generally due to extension from the tubes, and some observers have thought that removal of the appendages with curetting of the uterus is sufficient in these cases. Dr. T. S. Cullen, the writer of the report, however, advises removal of a portion of the uterus too in cases where tuberculous tubes are found; for the disease is generally limited to the upper part of the uterine body, which is not easily reached by the curette. The work is illustrated to show the various points in the pathology of the above conditions; several of the pictures are coloured, and all are highly finished. The printing is clear and very free from typographical errors.

**Edinburgh Hospital Reports.** Volume Third. Edinburgh: Young J. Pentland. 1895.—This volume fully maintains the reputation gained by the two previous ones as being amongst the very best of the hospital reports published. It contains a number of interesting papers of much scientific value on clinical subjects, or on matters closely connected with clinical observation. Without selecting especial papers, we may say that every medical man who reads these reports will find much to interest and instruct him. The illustrations are numerous and good, and the printing and appearance of the book do credit to the publisher.

**Transactions of the New York Academy of Medicine.** Second Series. Vol. X. for 1893. Printed for the Academy. 1894.—This volume gives good evidence of the vigorous life of the profession in New York, in spite of the death of one of its most distinguished ornaments, Dr. James R. Leaming, whose memorial address by Dr. J. Leonard Corning contains the following concluding passages:—"To me he seemed like an exceptional and beautiful creation—as are all great and good men, to remind us of the immeasurable possibilities of human nature. Such attributes of the spirit cannot die; for though the physical casket in which they were enshrined for a season may pass away, their essence shall remain forever in our hearts as a glowing memory." The paper by Murphy on "Intestinal Approximation" may especially be mentioned as one of the most advanced of the series.

**Proceedings of the West London Medico-Chirurgical Society.** Volume the Sixth. London: Baillière, Tindall and Cox. 1895. —This volume comprises the proceedings of the eleventh and twelfth sessions, 1892-93 and 1893-94, and the Cavendish lectures by Mr. Henry Morris and Sir William Broadbent, the latter on the treatment of typhoid fever, which subject is also dealt with in an interesting address by Dr. Donald W. C. Hood. It is a matter for congratulation that the book is probably the last of this series of tardily-appearing volumes, which, we presume, will be superseded by the Society's quarterly periodical, noticed on another page.

**Transactions of the Obstetrical Society of London.** Vol. XXXVI. London: Longmans, Green, and Co. 1895.—This volume contains a number of interesting papers, and will be found well worth perusal by all who are interested in obstetric work. In a paper on "Cases of Pregnancy and Labour with

Bright's Disease," Dr. Herman makes some important observations and deductions, amongst other things showing that the prognosis is worse in those cases in which the average daily excretion of urea falls much below 250 grains than in those in which it is higher; a rapid rise in the average amount of urea excreted daily after delivery is a favourable sign, as is also a marked increase in the quantity of urine. Dr. Harris, in "A Plea for the Practice of Symphysiotomy," gives an interesting series of cases, but fails to carry conviction to the minds of those who heard the paper: there is little doubt but that the objections to the operation in English practice are many, and that it is unlikely to have a wide application as a substitute for either craniotomy or Cæsarean section. Dr. Gow, in a paper on the "Relation of Heart Disease to Menstruation," appears to confute the opinion often expressed as to the prevalence of menorrhagia in valvular disease. Dr. Amand Routh, writing on "Associated Parovarian and Vaginal Cysts," throws considerable light on some rather obscure cases where tumours apparently parovarian in nature suddenly disappear, often with the occurrence of a profuse watery vaginal discharge. He attributes these cases to the emptying of the tumour through a persistent duct of Gartner. Dr. Giles makes some interesting observations on the relation between temperature after delivery and the duration of labour. Dr. Griffith cites a case which shows that the expulsion of a decidual cast cannot be considered good evidence of the existence of extra-uterine pregnancy. Many noteworthy specimens are described in detail, and much interesting matter will be found in the volume in addition to that mentioned. The general get up of the volume is as good as usual, and the printing of several charts, showing in graphic form the main points of interest, makes the papers very easy of digestion.

**The Transactions of the Edinburgh Obstetrical Society.** Vol. XIX. Edinburgh: Oliver and Boyd. 1894.—As the ground covered by these *Transactions* is large and the contributors are numerous, we cannot well discuss the many articles in detail. In a valedictory address by Professor A. R. Simpson we are reminded of the long-fought battle—concluded some thirty years ago—which was waged with such bitterness before the operation of ovariotomy was allowed a place in surgery; and Dr. Clay, the "father of ovariotomy" in England, is duly eulogised. Those interested in teratology will be pleased to see a continuation of Dr. Ballantyne's able articles on this subject, and here he treats of the anatomy of the different forms of the "amorphous" fœtus. We may here express our regret that Dr. Ballantyne's useful periodical *Teratologia* has not met with sufficient support to justify its continuance. We do not observe anything "new" in the various papers, but they are marked by a sound practical character, which is perhaps their chief feature, and which shows that the Society is well abreast of the times.

There is a useful index, and the printing of the book has been well done.

**Transactions of the American Dermatological Association.** New York: Geo. L. Goodman & Co. 1894.—We have read this volume with great pleasure, as it deals with very interesting and very rare matter. It is well got up, well printed, and well illustrated with drawings, photographs, and coloured plates. The first contribution is by Dr. G. T. Jackson on "Thyroid Feeding in Diseases of the Skin," and the conclusions arrived at by the author himself, and the members who entered into the discussion, are, generally speaking, these: That there is danger to the patient's life in giving any preparation of the thyroid gland, and that therefore the administration must be very carefully watched, and that in most cases of skin disease the good that has appeared might well be attributed to rest in bed and hospital attention, but that in most cases of myxœdema and cretinism the results are so good that the risk of dangers to the sufferers from these dire disorders may, justifiably, be run. "Cold as an Etiological Factor in Diseases of the Skin," by Dr. Wm. Thos. Corlett, of Cleveland, is a record of fourteen cases of an eruption, chiefly on the hands and forearms, caused by severe cold, and therefore occurring mostly in the winter, and hence named dermatitis hiemalis. The paper is illustrated by a very well-drawn and coloured plate, and by smaller photographs. We must not omit to call attention to Dr. G. H. Fox's communication on "The Rare Forms of Alopecia," which gave rise to very conflicting views; and to "A Case of Favus of the Head and Body," recorded by Drs. J. Abbott Cantrell and Emanuel Stout. Dr. H. W. Stelwagon, dealing with "The Question of Contagiousness of Molluscum Contagiosum," quotes more cases than any other collector whom we can remember. Dr. S. Sherwell describes a very remarkable case of "Icthyosis Congenita (so-called Harlequin Fœtus)." Dr. Edward B. Bronson's case of "Symmetrical Cutaneous Atrophy of the Extremities" is probably unique.

**Transactions of the Medical Society of the State of New York.** Philadelphia: William J. Dornan. 1895.—**Transactions of the Michigan State Medical Society.** Vol. xix. Grand Rapids: Seymour & Muir Printing Co. 1895.—**Transactions of the Medical Society of New Jersey.** Newark: L. J. Hardham. 1895.—**Transactions of the Iowa State Medical Society.** Vol. xiii. Des Moines: The Kenyon Printing and Mfg. Co. 1895.—**Transactions of the Medical Association of the State of Alabama.** Montgomery: Brown Printing Co. 1895.—**Transactions of the Ohio State Medical Society.** Toledo: The Blade Printing and Paper Co. 1895.—**Transactions of the Texas State Medical Association.** Galveston: Knapp Brothers. 1895.—These volumes contain many papers of more than ephemeral interest to the members of the respective associations; they indicate a degree of healthy medical vitality extended over a wide area of the United States, and give much information of world wide interest interspersed with much which can only be of interest locally and individually. One of the most useful and practical papers is that by Dr. Benedict, of Buffalo, on auscultatory percussion and allied methods of physical diagnosis (*Tr. M. Soc. N.Y.*, p. 351). He endeavours to locate the boundaries of an organ by noting when the sound of percussion becomes suddenly feeble, as heard through the stethoscope. So long as both plexor and stethoscope are over the same organ the sound conveyed to the ear is peculiarly sharp and penetrating: as soon as either passes the limits of the organ the sound becomes dull and diffused. The finger or a tuning-fork are the most convenient methods of instituting vibrations.

**Annual of the Universal Medical Sciences.** Five Volumes. Philadelphia: The F. A. Davis Company. London: F. J. Rebman. 1895.—The eighth issue of this *Annual* has arrived somewhat tardily towards the end of the year: it is difficult to keep a large editorial staff up to time, but the Editor speaks kindly of those who are responsible for the delay. We can only congratulate him on the result of his work, and wish that it may be long continued.

**The Year-Book of Treatment for 1896.** London: Cassell and Company, Limited.—This now well-known and always welcome annual has appeared somewhat earlier than usual, a fact which is greatly to the credit of the editor and the enterprising publishers, inasmuch as it has not been suffered to deteriorate, but worthily maintains the reputation of its predecessors. While, as always, it is a very useful volume for the busy practitioner, its sections vary considerably in scope and completeness; for instance, it is surprising, and we think a misfortune, that no allusion is made to the extremely important recent contributions on malignant disease of the larynx, which have entirely altered our position in regard to the radical treatment of a considerable proportion of these hitherto hopeless cases. It would be easy to add other examples of serious omission, while the insertion of less important matter, though doubtless useful and interesting, prevents our ascribing all these defects to the exigencies of space. Yet, with these few exceptions, the *Year-book* may be said to be a fairly complete and able *resumé* of the past year's therapeutical advance, and one that should be in the hands of every practitioner.

**Clinical Sketches.** London: Smith, Elder, & Co. 1895.—We congratulate Mr. Noble Smith on the result of his first year's editorial work, and on the fact that he will in future be able to continue a new series of this periodical at half the previous price. The December number of this journal concludes a series forming a very handsome volume of sketches, both artistic and literary. It contains a copy of the well-known portrait of John Hunter, painted by Sir Joshua Reynolds and engraved by Sharpe.

**The West London Medical Journal.** Published quarterly under the auspices of the West London Medico-Chirurgical Society. Vol. I., No. 1. January, 1896. London: John Bale & Sons.—We cannot but congratulate the promoters of this new quarterly on the appearance and dress of their *Journal*, which so much resembles our own. We quite think that a journal of this kind must add to the prosperity of the Society which undertakes it, and we wish both the *Journal* and the Society a useful and protracted life—with a continuance of the healthy vitality given to its first number by Dr. Symons Eccles on the advantages of oxidation.

**Pediatrics.** Vol. I. No. 1. January 1st, 1896. New York: Van Publishing Co.—This periodical, devoted to the diseases of children, is to be published semi-monthly. An editorial staff, composed of British and United States doctors, has been obtained. We congratulate the editor, Dr. George Carpenter, on his first number, which is not only full of good material but is well and artistically printed. We wish prosperity for the new journal, which seems to be an American publication with an English editor.

**Journal de Neurologie & d' Hypnologie.** Brussels: 3 Rue des Hirondelles.—This journal, the first number of which was issued in December, is to appear twice monthly. It is intended not only for neurologists, but to enable medical practitioners generally to keep themselves in touch with the advances made in neurology. Besides original articles, the journal will contain a general review on some subject of current interest in neurology or hypnotism, and short abstracts of cognate subjects. The names of the collaborators are a sufficient guarantee that the journal will be well written. The early numbers contain some interesting papers. We would suggest that the titles of the abstracts of foreign papers should not be translated into French, but given in the language in which they were written in order to facilitate reference to the original papers. If thought necessary, a translation of the title could be added.

**A.B.C. Medical Diary.** 1896. **A.B.C. Chemist's Vest-Pocket Diary.** 1896. London: Burroughs, Wellcome & Co.—We need only mention the titles of these products of "the firm that did." The *Medical Diary* is bound up with 180 pages of valuable information called the "Excerpta Therapeutica," the diary itself is of the ordinary weekly type. They both contain abstracts of the history of sero-therapeutics, therapeutic notes on the newer remedies, and a large number of useful tables for reference.

**The Professional Nurses' Diary and Ready Reference Book.** 1896. London: Burroughs, Wellcome & Co.—This is an exceedingly useful manual, consisting of 114 pages of serviceable diary matter prefaced by 78 pages of well-planned and well-written introduction, which deals amongst others with such subjects as "Nursing: Ancient and Modern," "General Principles of Nursing," "First Aid in Emergencies," "Foods and Feeding." If, as was to be expected, the preparations of the firm publishing this book are brought specially into notice, the value of the book is not thereby diminished, as most, if not all, of their preparations have stood the test of experience. Every nurse would be the better for adding the little work to her equipment.

**The English Illustrated Magazine.** London: *Illustrated London News* Office.—The March number is excellent. Amongst its articles is an interesting and well-illustrated one on "The New Light," dealing with Professor Röntgen's recent discovery. Doctors will also read with interest some "Minor Memories of Lord Leighton," of whom it is said that "the son and grandson of physicians, he found in anatomy a study which he described as 'fascinating.'"

